# Benchmarking of the quantification approaches for the non-targeted screening of micropollutants and their transformation products in groundwater

**DOI:** 10.1007/s00216-020-03109-2

**Published:** 2021-01-27

**Authors:** Anneli Kruve, Karin Kiefer, Juliane Hollender

**Affiliations:** 1 Department of Materials and Environmental Chemistry, Stockholm University, 106 91 Stockholm, Sweden; 2 Eawag: Swiss Federal Institute of Aquatic Science and Technology, 8600 Dübendorf, Switzerland; 3 Institute of Biogeochemistry and Pollutant Dynamics, ETH, 8092 Zürich, Switzerland

**Keywords:** LC/HRMS, Non-targeted, Quantification, Transformation products

## Abstract

A wide range of micropollutants can be monitored with non-targeted screening; however, the quantification of the newly discovered compounds is challenging. Transformation products (TPs) are especially problematic because analytical standards are rarely available. Here, we compared three quantification approaches for non-target compounds that do not require the availability of analytical standards. The comparison is based on a unique set of concentration data for 341 compounds, mainly pesticides, pharmaceuticals, and their TPs in 31 groundwater samples from Switzerland. The best accuracy was observed with the predicted ionization efficiency-based quantification, the mean error of concentration prediction for the groundwater samples was a factor of 1.8, and all of the 74 micropollutants detected in the groundwater were quantified with an error less than a factor of 10. The quantification of TPs with the parent compounds had significantly lower accuracy (mean error of a factor of 3.8) and could only be applied to a fraction of the detected compounds, while the mean performance (mean error of a factor of 3.2) of the closest eluting standard approach was similar to the parent compound approach.

## Introduction

Hundreds to thousands of micropollutants and their TPs in the water cycle are relevant from the ecotoxicology point of view as well as for water purification. This includes pesticides, pharmaceuticals, personal care products, and industrial contaminants. Pesticides are used to protect crops in farming across agriculture and reach the environment due to leaching and run-off [1–5]. Pharmaceuticals and personal care products result from sewage and are widespread micropollutants [1]. In the environment, pesticides, pharmaceuticals, and several other types of micropollutants degrade by biotic and abiotic processes to their transformation products (TPs). Additionally, chemical degradation through ozonation or chlorination may occur in water treatment plants [6]. Mostly, the toxicity of the TPs is similar or lower than that of the parent compound; however, cases, where the transformation products are more toxic than the parent compound, may occur [7, 8]. The final potential for an adverse effect of the water sample depends on the toxicology endpoints, bioaccumulation, and concentration of the micropollutants and their TPs [8]. Genuinely, the drinking water standard of 100 ng/L applies to pesticides and their relevant TPs in the European Union [9]. TPs are considered relevant if it is assumed that they still possess pesticidal properties or show severe and unacceptable toxicological effects [10].

The monitoring of all these micropollutants is unfeasible with conventional targeted analysis methods. Therefore, liquid chromatography high-resolution mass spectrometry (LC/HRMS)-based suspected and non-targeted screening is increasingly used to detect micropollutants, including TPs [11, 12]. In suspected and non-targeted screening, the compounds are first tentatively identified based on the exact mass of the compound, isotope pattern, predicted retention time, MS/MS spectra, etc. [13]. The full identification and quantification of the detected contaminants require confirmation with the analytical standards, which is often complicated due to their unavailability. Therefore, only a small fraction of the compounds detected can be identified at the highest confidence level and subsequently quantified. In order to manage the time-consuming identification challenge, tentatively identified compounds need to be prioritized before the final identity confirmation with purchased, partially expensive standards. One of the prioritization criteria for identification is the estimated concentration (intensity) of the contaminant.

The quantification of the detected micropollutants traditionally relies on the calibration graph approach, which assumes the availability of the analytical standards. In cases where analytical standards are not available, it is more complex to estimate the concentration of these compounds as the response factor differs from compound to compound in LC/ESI/HRMS. Differences as large as a factor of 1 million have been observed [14]. Therefore, alternative quantification approaches are of importance. The ones of particular interest to the quantification of micropollutants and corresponding transformation products are (1) using the response factor of the parent compound for quantification of the TPs, (2) using the response factor of internal standards eluting closest to the detected micropollutant [15], and (3) using the predicted ionization efficiency of the compound of interest [16].

The first approach has been developed to overcome the lack of analytical standards for TPs of pesticides and pharmaceuticals. It is assumed that the TPs have the same response as the parent compounds, which is based on the structural similarity of the TP and parent compound. This is based on the assumption that the structural changes occurring during the biotic or abiotic transformation of the parent compound do not result in significant structural modifications [17]. Especially, modifications related to the ionization behavior of the compounds such as basicity and hydrophobicity are assumed to be minimal.

The second approach, proposed by Pieke et al. [15], uses a set of internal standards spiked into the samples and uses the response factor of the internal standard eluting closest to the compound of interest for the quantification. This approach is based on the assumption that compounds eluting close to each other also have similar response factor, which further results from the assumption that the retention behavior in LC and ionization in ESI are influenced by the same properties of the compounds. This is likely to be true to some extent as both retention in reversed-phase chromatography [18, 19] and ionization efficiency in ESI [20–22] have been related to the hydrophobicity, expressed as log*P*, of the compound. However, both retention and ionization in ESI [23] are more complex processes and depend also on other factors such as acid-base properties and eluent properties.

The third approach aiming to estimate the concentration of the micropollutants without analytical standards is based on predicting the ionization efficiency of the compounds in the ESI source [16, 24–26]. In this approach, first, the ionization efficiency of the compound of interest is predicted from structural and eluent descriptors. Then, the ionization efficiency is converted into a response factor corresponding to the analysis conditions, including the instrument, using few calibration compounds that were measured at different concentration levels with the same LC/HRMS method. This approach aims to account for the effect of the structure of the compound, eluent composition, and instrument parameters (such as source geometry) on the response factor. The ionization efficiency-based quantification has been recently tested for quantification of pesticides in food samples [27].

The objective of this paper is to compare these three approaches described above for the quantification of 341 micropollutants analyzed with LC/ESI/HRMS in positive ionization mode. The comparison is based on an extensive dataset from 31 Swiss groundwater samples that especially focusses on pesticides and their TPs. The 74 micropollutants finally detected in the groundwater samples were previously robustly quantified from groundwater samples with the aid of a unique set of analytical standards. Therefore, this dataset is highly suitable for benchmarking different quantification approaches.

## Materials and methods

### Dataset

The samples originated from 31 groundwater monitoring sites in Switzerland. Most monitoring sites were located in agriculturally intensively used and/or densely populated areas. Dissolved organic carbon content was low (< 0.5–1.8 mg/L, median: 0.8 mg/L) and electrical conductivity as sum parameter for salt content ranged from 360 to 1000 μS/cm.

The samples were analyzed for 519 micropollutants (236 pesticides and TPs, 221 pharmaceuticals and TPs, 62 other compounds from urban sources) using LC/HRMS/MS. For quantification, 22 calibration standards (0.1–1000 ng/L prepared in ultrapure water; 0.1–2000 ng/L for chloridazon-desphenyl due to detections > 1000 ng/L), six spiked samples (analyte spiking level 10 ng/L or 100 ng/L), and the 31 samples were spiked with 224 isotope-labeled internal standards (ISTDs) at 100 ng/L. As micropollutant concentrations in groundwater are usually low, the samples, spiked samples, and calibration standards were enriched via vacuum-assisted evaporation [28] by a factor of 150 resulting in limits of quantification of ≤ 10 ng/L for 78% of the compounds. The concentrates were injected on a reversed-phase C18 column (Atlantis T3 3μm, 3.0 × 150 mm, Waters, Ireland; injection volume: 100 μL). Analytes were separated by gradient elution with water and methanol as eluents (both modified with 0.1% formic acid), ionized in electrospray (4/− 3 kV), and detected on an orbitrap mass spectrometer with a resolution of 140k in MS1 full-scan mode and data-dependent MS/MS acquisition (Q Exactive Plus, Thermo Fisher Scientific, USA). The compounds were quantified based on the peak area ratio of analyte and the assigned ISTD using TraceFinder 4.1 (Thermo Fisher Scientific, USA). A total of 142 analytes were quantified with structurally identical ISTD. If a structurally identical ISTD was not available, the ISTD was selected that coeluted with the analyte (± 2 min) and resulted in the best relative recovery (i.e., ideally close to 100% and low deviation across spiked samples) using an in-house R script [29].

Finally, a total of 62 pesticides and TPs, 22 pharmaceuticals and TPs, and 19 other compounds from urban sources were detected in the groundwater samples at concentrations ranging from 0.2 to 1800 ng/L. Individual samples contained four to 44 compounds. For details on sample preparation, analysis, quantification, and final concentrations, see Kiefer et al. [2].

### Compound selection

For this study, we limited our analysis to compounds ionizing in positive ionization mode as much more compounds could be quantified in this mode. Compounds quantified using the sodium or ammonium adducts were removed from the dataset, due to the fact that currently ionization efficiency (approach III) for adducts cannot be predicted; however, if both protonated molecules and adducts are formed, the ionization efficiency can be predicted for the protonated molecule and the compounds can be quantified. Additionally, compounds that could neither be separated by chromatography nor *m/z* (in Kiefer et al. [2], the concentration sum of these compounds was reported) and compounds that could only be quantified with high uncertainty as no suitable ISTD was available (relative recovery < 50% or > 150% or relative standard deviation of relative recoveries in spiked samples > 50%) were removed so that the final dataset consisted of 341 compounds.

### Ionization efficiency predictions

The ionization efficiency values were predicted with a random forest regression that has been recently developed by Liigand et al. [16] based on 3139 data points in ESI+ mode and improved by adding further data. The solvent parameters were ramped over a wider range: pH 1.8 to 10.7, organic modifier content 0 to 100%. In addition, both methanol and acetonitrile as well as various different buffers were used. Experimental design was used to cover a wide range of possible solvent combinations. All ionization efficiency measurements were carried out in the linear range. Also, eight different instruments with different ionization source design were used in the measurements, starting from simple triple quadrupole and ion trap instruments to ion mobility/time of flight and Orbitrap mass spectrometers. To describe the compound structure, PaDEL 2D descriptors were used. The eluent composition is described with five empirical eluent descriptors: viscosity, surface tension, polarity index, pH, NH_4_^+^ content (yes/no). Accordingly, the effect of eluent composition is accounted for in the ionization efficiency predictions but the impact of the instrumental parameters used for measurements is not. Therefore, the predicted ionization efficiency values need to be converted into instrument-specific response factors as described below.

### Response factor prediction

For calibration of the predicted ionization efficiency values and the quantification based on the closest eluting calibration standard, the following 20 compounds were used: asulam, carbendazim, chloridazon, darunavir, disopyramide, exemestane, fenamidone, ifosfamide, melamine, mepivacaine, mesosulfuron-methyl, metosulam, monolinuron, monuron, N-(4-aminophenyl)-N-methyl-acetamid, pregabalin, primidone, sulfadiazine, thiacloprid, trimethoprim. The compounds were selected by randomly sampling 20 compounds from the list of all investigated compounds. The sampling was repeated three times and was performed in R with *sample.split* function from the caTools package [30]. The set with the widest range of retention time and response factors was chosen for the final quantification approaches. Later, these compounds are referred to as calibration compounds.

The peak area of the analytes in standards and samples was first corrected with the internal standard signal, if not specified otherwise:

1 $$ {\mathrm{Signal}}_{\mathrm{sample}}^{\mathrm{analyte},\mathrm{corrected}}={\mathrm{Signal}}_{\mathrm{sample}}^{\mathrm{analyte}}\bullet \frac{\mathrm{Mean}{\mathrm{Signal}}_{\mathrm{standard}}^{\mathrm{ISTD}}}{{\mathrm{Signal}}_{\mathrm{sample}}^{\mathrm{ISTD}}} $$

where ${\mathrm{Signal}}_{\mathrm{sample}}^{\mathrm{analyte},\mathrm{corrected}}$ is the corrected signal for a specific compound in either sample or standard; ${\mathrm{Signal}}_{\mathrm{sample}}^{\mathrm{analyte}}$ is the not corrected signal for a specific compound in the sample or standard; ${\mathrm{Signal}}_{\mathrm{sample}}^{\mathrm{ISTD}}$ is the signal of the internal standard in the respective sample or standard; and $\mathrm{Mean}{\mathrm{Signal}}_{\mathrm{standard}}^{\mathrm{ISTD}}$ is the mean signal of the same internal standard in all of the measured standards. In addition, all signals were corrected for the isotope peaks by multiplying the monoisotopic peak area with the isotope correction factor. This is necessary as the ionization efficiency predictions assume that all of the gas-phase ions, including isotope peaks and in-source fragments, formed from the protonated molecules are summed up.

The response factors RF of all of the compounds in the standard solutions were calculated as follows:

2 $$ \mathrm{RF}=\frac{{\mathrm{Signal}}_{\mathrm{standard}}^{\mathrm{analyte},\mathrm{corrected}}}{c(M)} $$

assuming that the intercept of the calibration graph is negligible and thereafter averaged.

### Concentration prediction

For concentration prediction, three quantification approaches were used:

I. Using the response factor of the parent compound to quantify the TPs.

3 $$ {c}_{\mathrm{TP}}=\frac{{\mathrm{Signal}}^{\mathrm{analyte},\mathrm{corrected}}}{{\mathrm{RF}}_{\mathrm{parent}\ \mathrm{compound}}} $$

II. Using the response factor of the calibration compound eluting closest to the compound of interest.

4 $$ {c}_{\mathrm{sample}}^{\mathrm{analyte}}=\frac{{\mathrm{Signal}}_{\mathrm{sample}}^{\mathrm{analyte},\mathrm{corrected}}}{{\mathrm{RF}}_{\mathrm{sample}}^{\mathrm{calibration}\ \mathrm{compound}\ \mathrm{eluting}\ \mathrm{next}\ \mathrm{to}\ \mathrm{the}\ \mathrm{compound}}} $$

III. Predicting the ionization efficiency of the compound and using this for quantification. The ionization efficiency values were predicted with a previously published model [16] that has been further improved by adding new data to the model training. As the model output is in universal ionization efficiency values and not instrumentation specific, the response factors of the 20 abovementioned calibration compounds were used to transform the ionization efficiency values to instrumentation-specific response factors. For this, the RF values of these 20 calibration compounds were correlated with the predicted ionization efficiency values logIE_pred_:

5 $$ \log \mathrm{RF}=\mathrm{Slope}\bullet \log \mathrm{I}{\mathrm{E}}_{\mathrm{pred}}+\mathrm{intercept} $$

The logarithmic scale with base 10 was used to have even weight on all of the points as the values range over several orders of magnitude.

Thereafter, the slope and intercept were used to transfer the ionization efficiency values predicted for the other compounds to the response factors. The concentrations were calculated as follows:

6 $$ {c}_{\mathrm{sample}}^{\mathrm{analyte}}=\frac{{\mathrm{Signal}}_{\mathrm{sample}}^{\mathrm{analyte},\mathrm{corrected}}}{{\mathrm{RF}}_{\mathrm{predicted}}} $$

We express the accuracy of the predicted concentrations as a fold base prediction error

7 $$ \mathrm{prediction}\ \mathrm{error}=\max \left\{\begin{array}{c}\frac{\mathrm{predicted}\ c}{\mathrm{measured}\ c}\\ {}\frac{\mathrm{measured}\ c}{\mathrm{predicted}\ c}\end{array}\right. $$

The relative error was used to be able to compare the prediction accuracies for low and high concentrations as well as to treat equally the over- and underestimation.

The code and data are available from https://github.com/kruvelab/semi-quantification-water-micropollutants.

## Results

**Fig. 1:**
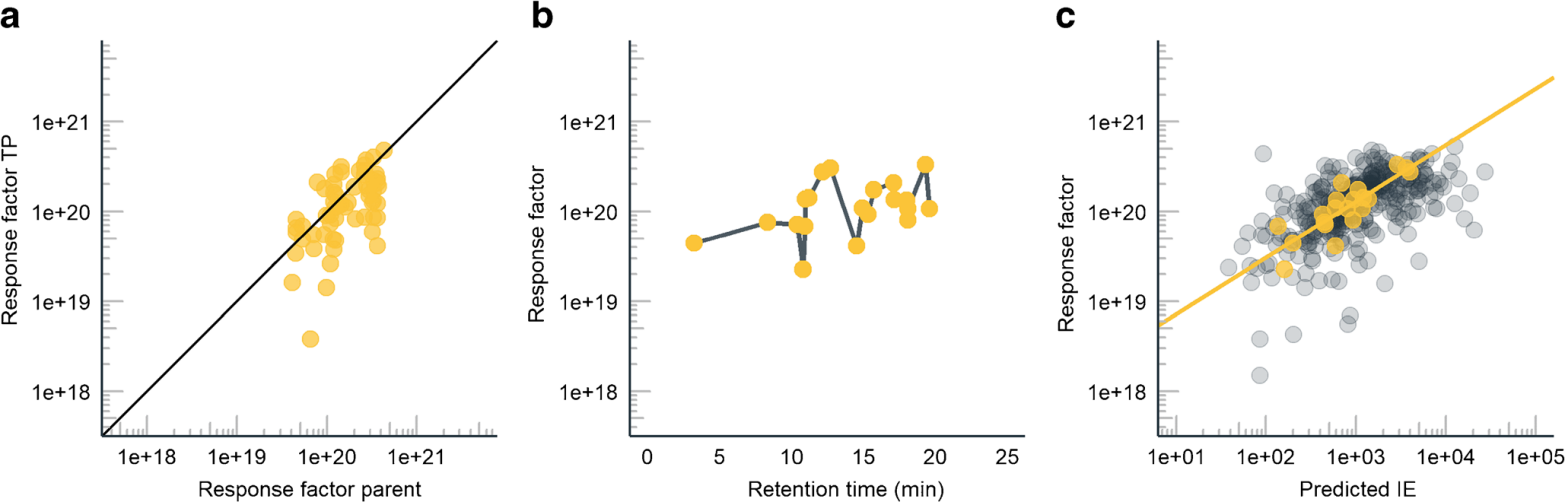
RF factor, averaged over all concentrations of the standard, as a function of relevant parameters used in the different approaches. (**a**) The comparison of the response factor for parent and transformation product. The line shows an ideal case where the response factor of TP and parent are equal. (**b**) The response factor of the calibration compounds as a function of the retention time. The compounds with 2 lowest response factors are asulam and primidone. (**c**) The agreement of response factor predicted based on the ionization efficiencies vs the measured response factor, yellow dots show the calibration compounds used for transferring ionization efficiency values to instrument-specific response factors

Altogether, 341 compounds were included in the dataset, out of these 20 compounds were used as calibration compounds for approach II and for transferring ionization efficiency values for approach III. The calibration compounds were sampled randomly to cover a wide retention time range: from 3.2 to 19.6 min and response factors from 2.2·10^19^ to 3.3·10^20^ (M^−1^) while the corresponding ranges for the whole dataset were 3.2 to 22.1 min and 1.5·10^18^ to 5.3·10^20^ (M^−1^). The coverage of the retention time and response factor range is visualized in Fig. 1.

The concentration of the calibration graph solutions ranged from 5 × 10^−13^ to 1 × 10^−8^ M (0.1–1000 ng/L) and a linearity check was used to avoid data points lower than the quantification limit or higher than the upper limit of linearity. For groundwater samples, the concentrations included in the dataset ranged from 5 × 10^−13^ to 1 × 10^−8^ M.

**Fig. 2:**
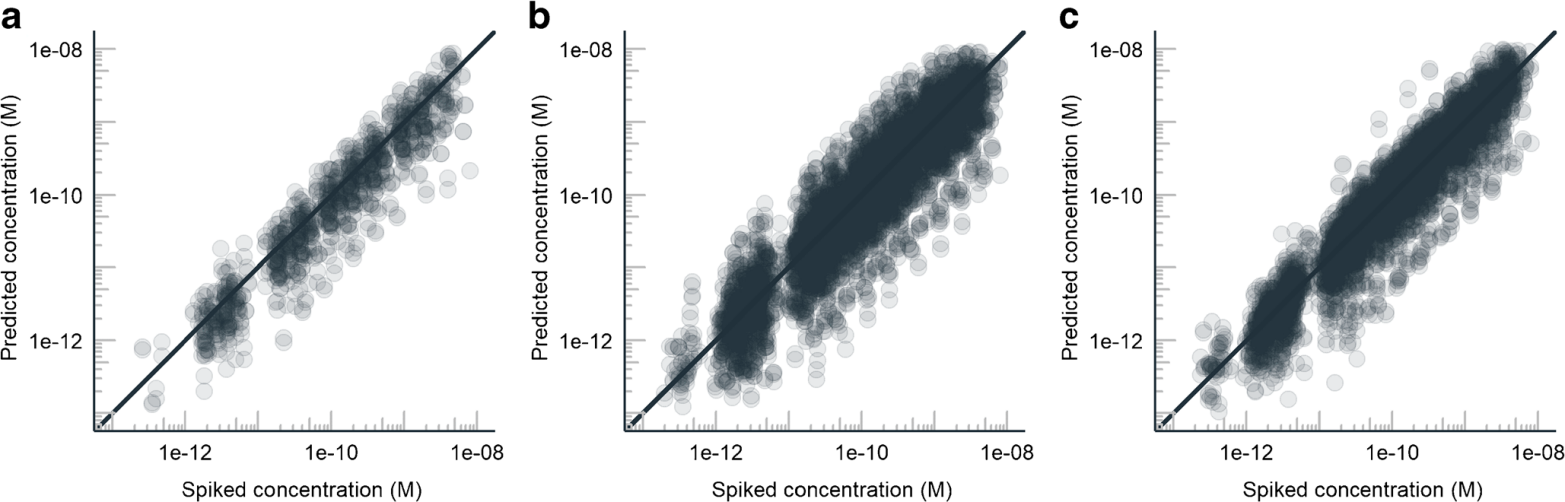
The predicted concentrations vs spiked concentrations for the standard: (**a**) using the response factor of the parent compound for quantification; (**b**) using the response factor of a close eluting standard; and (**c**) using response factor based on the predicted ionization efficiency. The number of data points including all 341 compounds over various concentrations is 5824. The exception is the first approach (1135 data points), using the response factor of the parent compound for quantification of the TP, as this approach is applicable only for TPs

**Fig. 3:**
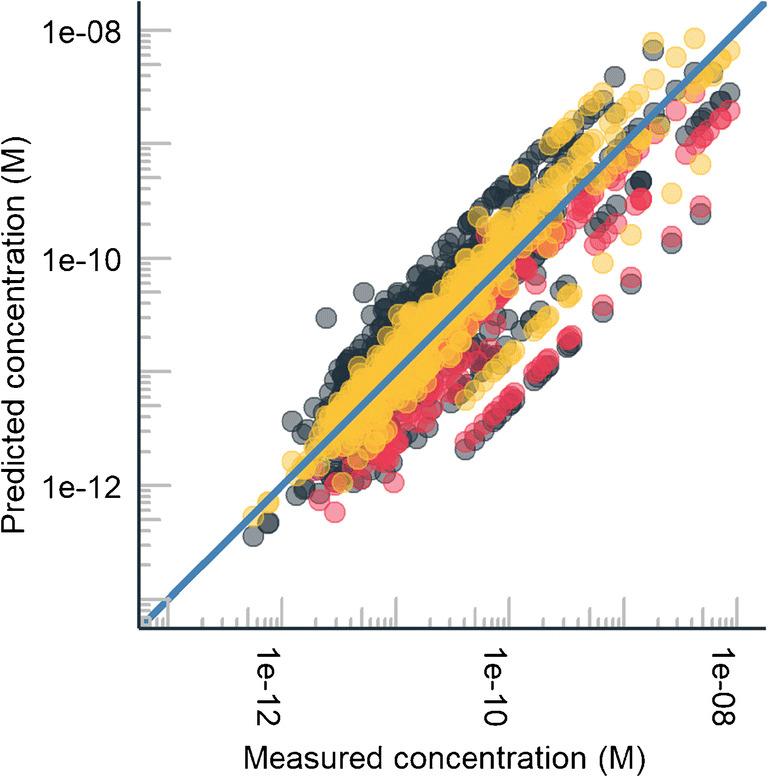
The predicted concentrations for micropollutants with the three approaches in the 31 groundwater samples. Pink dots correspond to the approach where the TPs were quantified based on the response factor of the parent compound; the mean prediction error was 3.8×. Gray dots correspond to the approach where the calibration graph of the compound closest to the analyte was used for quantification; the mean prediction error was 3.2×. Yellow dots correspond to the approach where ionization efficiency of the compound was used to predict the response of the compounds; the average error was 1.8×. With all approaches, the peak areas of the compounds were first corrected with the response of the ISTD according to Eq. 1. The diagonal line shows the perfect fit

On average, the highest prediction accuracy both for standards (Fig. 2) and real samples (Fig. 3) was observed for the ionization efficiency-based quantification approach. Though small numerical differences between ISTD corrected and not corrected results exist, the differences are not statistically significant for any of the quantification methods. Also, the differences between standards and spiked samples are statistically insignificant within one quantification approach. The comparison of standards and real samples is complicated as not all micropollutants and TPs were detected in the samples. For real samples, the lowest prediction accuracy was observed for the parent compound-based approach, which is restricted to TPs. The mean error was a factor of 2.1 and 1.8 with ionization efficiency-based quantification for standards and samples, respectively. The summary of the performance of the three approaches is given in Table 1.

**Table 1:** The summary of the performance of the quantification approaches for standards, spiked samples, and real samples. The results are presented both for ISTD corrected peak areas and not corrected peak areas

Quantification approach		RF_parent compound_		RF_standard eluting closest_				RF_predicted ionization efficiency_			
		ISTD corrected	No correction	ISTD corrected		No correction		ISTD corrected		No correction	
Standards	Number of compounds	60		341	60 TPs	341	60 TPs	341	60 TPs	341	60 TPs
	Mean error	2.5×	2.2×	3.0×	3.0×	3.3×	3.2×	2.1×	2.3×	2.0×	2.0×
	Max error	38×	17×	82×	44×	88×	20×	62×	22×	37×	11×
	% of datapoints with error < 10×	97%	98%	97%	98%	96%	96%	98%	98%	98%	99%
Spiked samples	Number of compounds	60		341	60 TPs	341	60 TPs	341	60 TPs	341	60 TPs
	Mean error	2.3×	2.5×	2.9×	2.9×	3.2×	3.2×	2.0×	2.1×	2.2×	2.3×
	Max error	17×	20×	59×	19×	61×	22×	51×	51×	61×	61×
	% of datapoints with error < 10×	98%	98%	97%	98%	97%	96%	98%	99%	98%	99%
Real samples	Number of compounds	23		74	23 TPs	72	23 TPs	74	23 TPs	72	23 TPs
	Mean error	3.8×	4.3×	3.2×	3.8×	3.6×	4.2×	1.8×	2.0×	1.8×	2.1×
	Max error	17×	20×	19×	19×	22×	22×	7×	7×	9×	9×
	% of datapoints with error < 10×	90%	89%	95%	90%	95%	90%	100%	100%	100%	100%

## Discussion

### Quantification with the parent compounds

In the current dataset, the parent compound could be used for the quantification of 60 transformation products. It was evaluated that TPs mostly had response factors lower than the parent (Fig. 1a). For example, N-N-dimethylsulfamide had a factor of 17 lower response factor than the corresponding parent compound, N-N-dimethyl-N-(4-methylphenyl)-sulfamide. This is expected, as the TP is significantly less hydrophobic than the parent (log*P* changes from 1.2 to − 0.9) due to the cleavage of a benzene ring. The hydrophobic moieties are very important to assure high ionization efficiency in ESI, as more hydrophobic compounds are more surface-active and partition to the surface of the ESI droplets, which facilitates the formation of gas-phase ions.

**Fig. 4:**
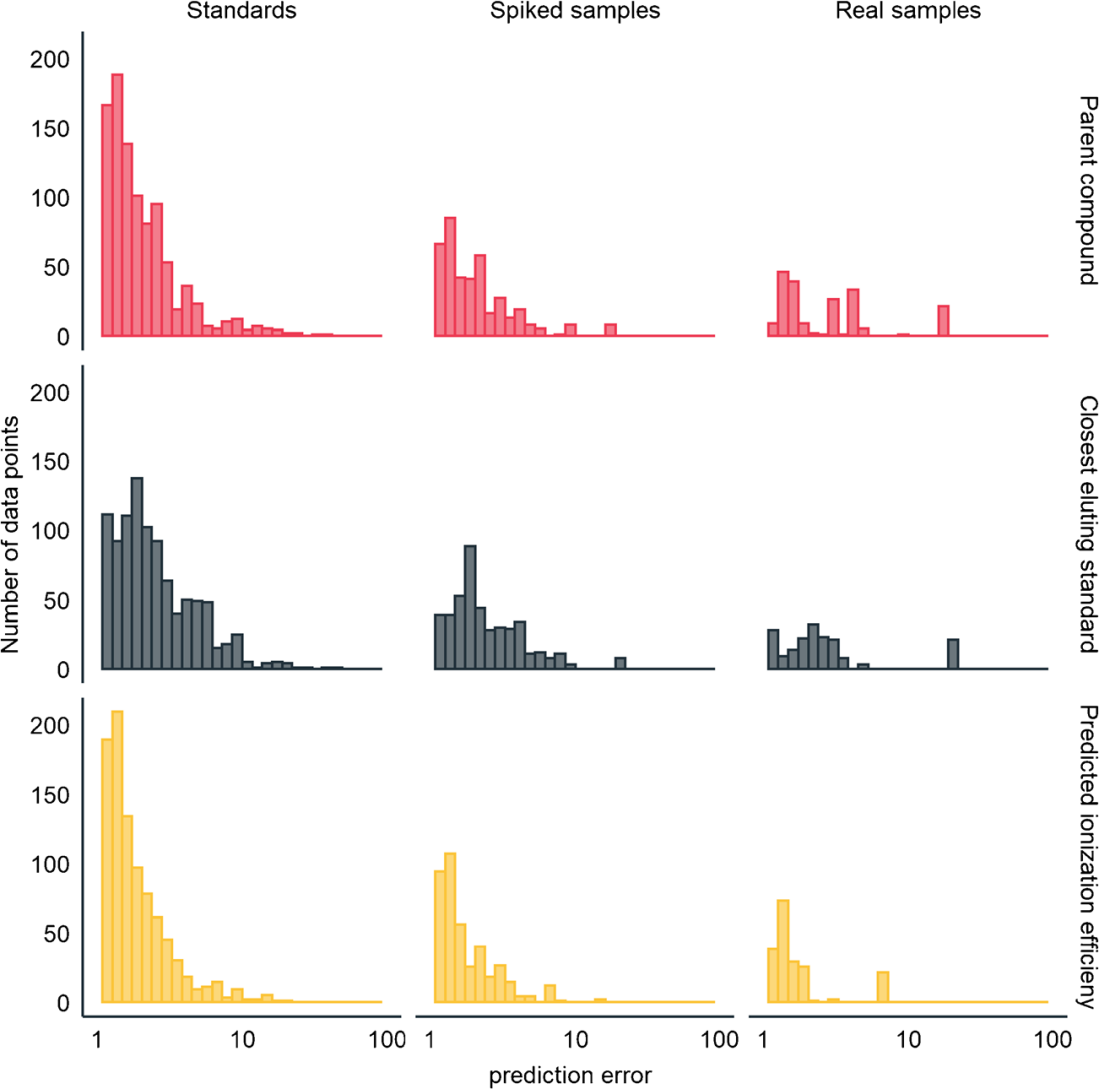
The distribution of the concentration prediction errors (in folds) for standards, spiked samples, and real samples with all quantification approaches

On average, this approach had intermediate performance for standards and the lowest performance for the real samples. The average concentration prediction accuracy for the standards and samples was a factor of 2.5 and 3.8, respectively. The maximum prediction error was a factor of 38 for N,N-dimethylsulfamide. The fraction of data points having an accuracy better than an order of magnitude was the lowest in comparison to other quantification approaches, 90% of all of the TPs detected in the samples. The error distribution for parent compound-based quantification (Fig. 4) is tailing towards higher errors than the error distribution for ionization efficiency-based quantification; however, it is much narrower than for the closest eluting standard-based quantification.

The parent compound approach also has a major disadvantage: it can only be applied if the compound to be quantified is a TP. Therefore, in this study, it could be applied to only 203 out of the 475 detected and identified peaks in the real samples. For a number of cases, the TP and parent also ionize in different ionization modes or one of them is not detectable with LC/HRMS and, therefore, the parent compound cannot be used for the quantification of the TP. For example, chlorothalonil is not detected in LC/ESI/HRMS while the corresponding TPs are well detectable.

### Quantification with the closest eluting standard

The retention behavior of a compound is influenced by the genuine hydrophobicity of the compound as well as the dissociation degree of the compound [31]. In reverse-phase chromatography, the compounds ionized at the given eluent conditions are eluting earlier than the respective compounds in their neutral form would.

At the same time, the ionization efficiency of the compounds in ESI is also influenced both by the hydrophobicity and acid-base properties of the compound [14, 20–22]. For example, in electrospray positive mode, the more hydrophobic compounds have higher ionization efficiency than the less hydrophobic ones in case of similar basicity [32]. Similarly, basic compounds that are protonated and possess a positive charge in the used eluent have higher ionization efficiency than the neutral compounds [33]. Therefore, the effect of acid-base properties of the compounds on the retention behavior in reversed-phase chromatography and ionization efficiency in ESI is different.

The closest eluting standard approach assumes a monotonic change in compounds’ ionization efficiency along the retention time. Additionally, the organic solvent content increases with increasing retention time further improving the ionization efficiency [33, 34]. If the acid-base properties of all of the studied compounds would be similar, the quantification based on close eluting standards would perform better. This has been previously shown for fatty acids by Kamga et al. [35]. However, the compounds in this study have very different acid-base properties. For example, we can see from Fig. 1b that the response factor of primidone (RT 14.5 min) is much lower than the ionization efficiency of the neighboring compound disopyramide (12.7 min). Primidone is a neutral compound in the mobile phase while disopyramide is strongly basic with several hydrophobic moieties. Due to the lack of significantly basic functional groups, primidone has a lower ionization efficiency and, accordingly, also a lower response factor in ESI positive mode. As a result, though these compounds are eluting next to each other as a combination of the hydrophobicity and acid-base properties, their ionization efficiencies are notoriously different.

The maximum difference in the response factor of the compound of interest and its closest eluting calibration compound was more than a factor of 60 for spironolactone, which is a late eluting compound that is not well ionizable in the mobile phase and has low ionization efficiency. Additionally, spironolactone can undergo significant in-source fragmentation. Ideally, the signals of the molecular ion and in-source fragments would need to be added up to account for all of the ions formed from. The corresponding calibration compound was fenamidone, a hydrophobic compound with low basicity. Therefore, using the response factor of the compound eluting closest to the compound of interest is resulting in a poor concentration prediction accuracy.

The closest eluting standard-based quantification showed an intermediate prediction accuracy compared to the other two approaches. The mean errors of the standard solutions and samples were a factor of 3.0 and 3.2, respectively. In the case of real sample analysis, 95% of the compounds had an error less than a factor of an order of magnitude and the maximal error was a factor of 19.

### IE prediction accuracy

Lastly, the ionization efficiency-based quantification was investigated. The agreement of the measured response factor and the predicted ionization efficiency is shown in Fig. 1c; the 20 compounds used for transferring the ionization efficiencies to response factors are shown in yellow.

**Fig. 5:**
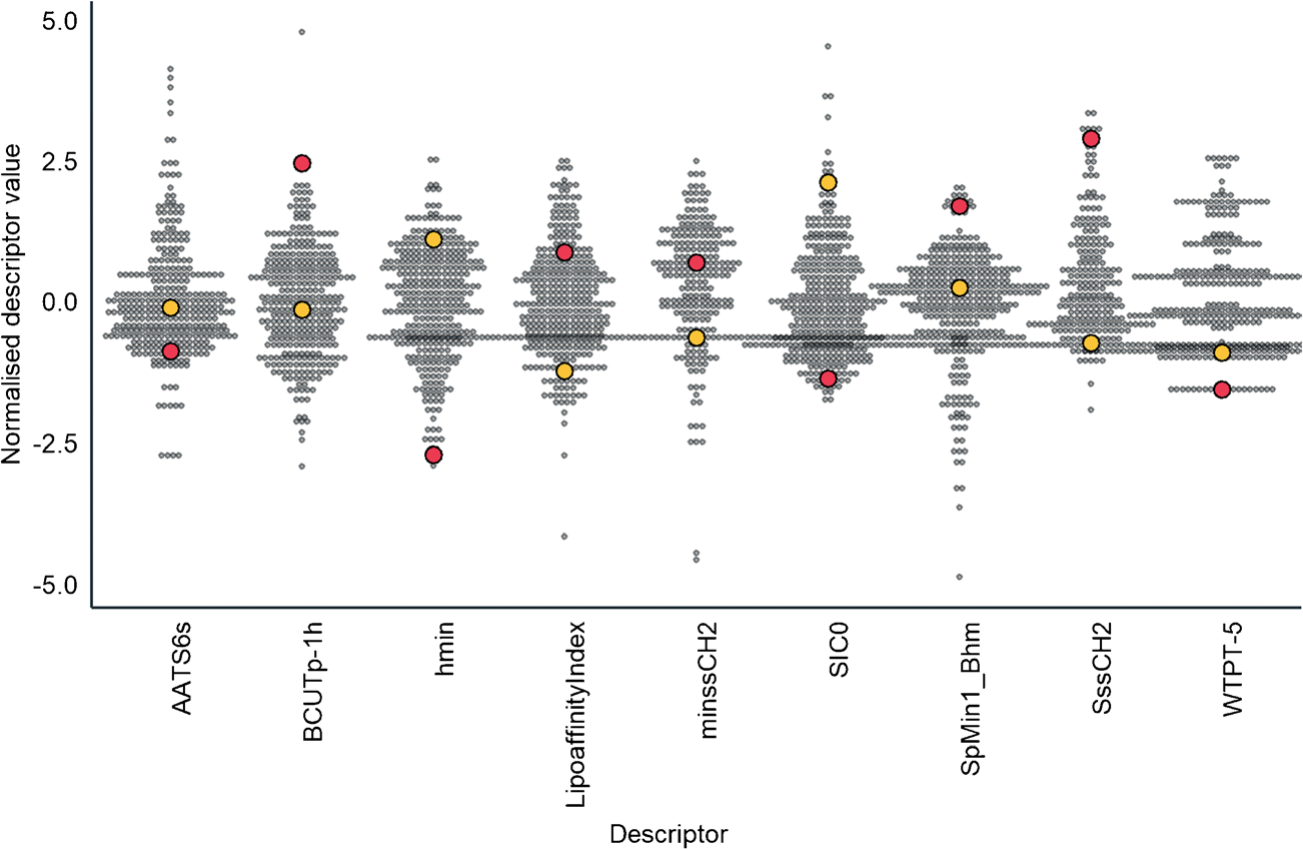
The distribution of the most important descriptors in the random forest algorithm for all compounds. Spironolactone and benzisothiazolin-3-one (BIT), two of the most poorly predicted compounds, are shown with red and yellow, respectively. Minimum and sum of CH_2_ atom type (minssCH2 and SssCH2), minimum H and LipoaffinityIndex are all related to the hydrophobic moieties of the compounds. Sum of path lengths starting from nitrogens (WTPT-5) describes the molecular branching while the rest of the parameters describe the topological properties of the molecule

On average, the agreement between the predicted and measured response factors was good (Fig. 1c). The major outliers where the predicted ionization efficiency was significantly higher than the observed response factor were spironolactone (21×), mono(2-acryloyloxyethyl)-succinate (19×), and methsuximide (17×). For benzisothiazolin-3-one (BIT) the predicted response factor was significantly lower (15×) than the observed RF. The analysis of the values of the most influential molecular descriptors in the random forest model [16] showed that the values for these compounds were within the range of expected descriptor values (Fig. 5). Therefore, the poor prediction accuracy likely results from missing structurally significant descriptors in the random forest model. This can occur if the variation in the values of these structural descriptors was not significant in the training set of the model. For example, spironolactone contains a thioester group, which might be important from the ionization efficiency point of view but is poorly represented in the dataset used for developing the random forest model for predicting ionization efficiency values. The same is likely to be true for the isothiazole group, present in benzisothiazolin-3-one (BIT) that is also poorly represented in the dataset used for model training. This is partially supported by the slight underestimation of the response factors for 5-chloro-2-methyl-4-isothiazolin-3-one (CMI) (3×) also containing the isothiazole group; however, for 2-n-octyl-4-isothiazolin-3-one (OIT) and 2-methyl-4-isothiazolin-3-one, overestimation was minimal. Therefore, it is very important to keep updating the ionization efficiency prediction model with new data and to improve the structural coverage of the training dataset.

The ionization efficiency-based quantification showed the highest accuracy and the narrowest error distribution (Fig. 4). The mean prediction error for standards (Fig. 2) and samples (Fig. 3) was a factor of 2.1 and 1.8, respectively. On average, the predictions errors were lowest with the ionization efficiency-based quantification; though, for some TPs, the parent compound-based quantification showed lower error. The parent-TP pairs cetirizin and 1-[(4-chlorophenyl)phenylmethyl]piperazin as well as normianserin and mianserin had more than 3× lower prediction error for TPs if the response factor of the parent compound was used, compared to the ionization efficiency-based quantification. This is also expected as these parent compound and TPs are very similar from the ionization efficiency point of view: during transformation, both the hydrophobic part of the compound and a basic center are retained.

### Prediction accuracy for real samples

The final accuracy of the quantification of the micropollutants was evaluated based on the results obtained from the real groundwater samples where altogether 74 pollutants were detected and quantified in positive ionization mode. The reference concentrations were obtained from the quantification with the analytical standards. The molar concentrations ranged over four orders of magnitude from 10^−13^ to 10^−8^ M (0.2 to 1800 ng/L). In general, the performance of the quantification approaches in real samples and standards was similar. However, for quantification with the parent compound, the mean prediction errors were a factor of 2.5 to 2.3 for standards and spiked samples, respectively (60 TPs in both cases). The mean prediction error for real samples was a factor of 3.8. However, only 23 TPs were detected in the real samples. While using the response factor of the closest eluting standard, a mean error of a factor of 3.0 (*n* = 60), 2.9 (*n* = 60), and 3.2 (*n* = 23) for standards, spiked samples, and samples, respectively, was observed. For ionization efficiency-based quantification, the mean prediction error was a factor of 2.3 (*n* = 60), 2.1 (*n* = 60), and 1.8 (*n* = 23). Consequently, the prediction accuracy was roughly the same for standards and spiked samples (same set of compounds) in case of all quantification approaches.

The trends from spiked samples to real samples are not directly comparable due to different numbers of compounds; however, comparison of the quantification approaches across real samples shows best accuracy for predicted ionization efficiency-based quantification. On the average, a factor of 2 lower prediction error has been observed with predicted ionization efficiency approach than with the other two approaches. The quantification accuracy in the spiked and real samples is influenced by several factors. Firstly, in samples, the compounds are coeluting with several matrix components that may cause ionization suppression, also called matrix effect [36]. The ionization suppression results in slightly more underestimated concentrations than overestimated concentrations for both parent compound and closest eluting standard-based quantification. The results of the parent compound and closest eluting standard-based quantification are more likely to be influenced by the matrix effect than the ionization efficiency-based quantification. The matrix effect varies from chromatographic peak to another depending exactly on the coeluting compounds. Additionally, even for two coeluting compounds, the magnitude of the matrix effect may be very different depending on the physicochemical properties of the compounds [34, 37, 38]. Therefore, it is likely that for these approaches the matrix effect is different for the compound of interest and the standard used for its quantification. On the other hand, ionization efficiency-based quantification does not apply the response factor of a single standard but calculates the ionization efficiency of each individual compound and then transfers these values to response factors with the aid of all 20 calibration compounds. Therefore, the sharp differences in matrix effect from peak to peak are partially cancelled out by using all 20 compounds for the transformation to the instrument conditions.

Secondly, the number of pollutants in real samples and standards differed (74 vs 341). This can cause random variation depending on the suitability of the quantification approaches for the particular set of compounds. For example, none of the compounds yielding low accuracy for standards was detected in the real samples. For a rigorous comparison, we evaluated separately the method performance for only the 23 TPs that could be quantified by all three approaches (see Table 1) from the groundwater samples. Though the mean and maximum prediction errors slightly change, the trends stay the same. The ionization efficiency-based quantification approach clearly outperforms the other two methods regarding all three performance criteria (mean error, maximum error, and percentage of data points with error less than a factor of 10).

The same trends are also seen for six spiked samples (see Table 1). The spiked concentrations were either 10 or 100 ng/L, depending on the sample. The mean concentration prediction errors were a factor of 2.3, 2.9, and 2.0 for parent compound-, closest eluting standard-, and ionization efficiency-based quantification, respectively. Therefore, we can conclude that the selection of the compounds does affect the performance of the approaches; however, even though the values of the performance characteristics somewhat alter from standards to spiked samples to real samples, the trends remain the same.

### Quantification without ISTD correction

All of the previously described results have been obtained from signals corrected with structurally identical or closest eluting ISTD; however, often such a wide set of ISTDs are not available. Therefore, it was of interest to compare the performance of the quantification approaches when no ISTD correction was applied. It was observed that the performance of the approaches was similar. For example, the mean prediction error for standard and samples with the ionization efficiency approach was a factor of 2.1 and 1.8, compared to a factor of 2.0 and 1.8 observed for ISTD corrected results. Similar results were also observed for other quantification results (see Table 1). Additionally, the trends across different quantification approaches were similar for both ISTD corrected and not corrected results. This demonstrates two important aspects; firstly, the comparison of the quantification approaches is robust as the results hold for both ISTD corrected and not corrected signals. Secondly, the matrix effect does not have a statistically significant impact on the performance of the quantification approaches. Additionally, it becomes obvious that the errors in the modelling are much larger than the errors in the analytical workflow that are corrected by the ISTDs.

## Conclusions

The major hurdles towards the unequivocal applicability of the non-targeted screening in decision-making include the lack of quantitative information obtained from the analysis if analytical standards are not available. Here, we have compared three approaches to enable a quantitative analysis of micropollutants in groundwater without the analytical standards. For both standards and real samples, the highest accuracy was observed with the ionization efficiency-based quantification. The mean quantification error was a factor of 2.1 and 1.8 for standards and real samples, respectively. The quantification errors were somewhat larger for approaches using the response factor of the parent compound for quantification of the transformation products (mean error 2.5 and 3.8) and the approach using the response factor of the closest eluting analytical standard (mean error 3.0 and 3.2). The parent compound-based quantification also suffers an obvious limitation in the application range.
